# Coping with extreme heat: current exposure and implications for the future

**DOI:** 10.1093/emph/eoae015

**Published:** 2024-08-22

**Authors:** Charles A Weitz

**Affiliations:** Department of Anthropology, Temple University, Philadelphia, PA 19122, USA

**Keywords:** heat stress, indoor heat, heat stress metrics, heat acclimatization

## Abstract

A preview of how effective behavioral, biological and technological responses might be in the future, when outdoor conditions will be at least 2°C hotter than current levels, is available today from studies of individuals already living in extreme heat. In areas where high temperatures are common—particularly those in the hot and humid tropics—several studies report that indoor temperatures in low-income housing can be significantly hotter than those outdoors. A case study indicates that daily indoor heat indexes in almost all the 123 slum dwellings monitored in Kolkata during the summer were above 41°C (106°F) for at least an hour. Economic constraints make it unlikely that technological fixes, such as air conditioners, will remedy conditions like these—now or in the future. People without access to air conditioning will have to rely on behavioral adjustments and/or biological/physiological acclimatization. One important unknown is whether individuals who have lived their entire lives in hot environments without air conditioning possess natural levels of acclimatization greater than those indicated by controlled laboratory studies. Answering questions about the future will require more studies of heat conditions experienced by individuals, more information on indoor versus outdoor heat conditions, and a greater understanding of the behavioral and biological adjustments made by people living today in extremely hot conditions.

## INTRODUCTION

Data generated by the world-wide use of standardized measurement instruments and practices (along with satellite-based technologies) leave no doubt that global temperatures have increased substantially over the past 40 years [1], leading to hotter summers [2] and more frequent and severe heat waves in many parts of the world [3, 4]. This increases the likelihood that heretofore implausible temperature extremes could occur globally [5]. The consequences are potentially dire. Several studies have predicted that intolerable heat in the future could increase excess mortality due to hyperthermia by more than an order of magnitude compared to current levels [6]. An important evolutionary implication of these predictions is that humans may be on the verge of reaching the adaptive limits of the physiological responses to the heat that have served our species so effectively for the past 200 000 years or so [7], and possibly earlier species of the genus *Homo* as well [8]. But modeling the mortality consequences of approaching this putative adaptive limit is complicated by the fact that humans may adjust behaviorally, biologically and technologically to extreme heat conditions [9, 10].

This short review explores the possibility that heat stress—particularly indoors in hot humid climates—may be greater than indicated by measurements made at outdoor weather stations and, therefore, may already be at levels predicted for the future. Three heat stress indices are briefly explained, and the heat tolerance limit associated with each one is used as a gauge against which current extreme heat exposure can be compared. The review concludes by highlighting some of the technical, behavioral and biological responses that people make to extreme heat, and the potential of these responses to provide meaningful adjustments to future heat increases.

## HEAT STRESS METRICS

An essential requirement for understanding how humans experience and respond to extreme heat is the identification of an appropriate metric to quantify heat stress. Perhaps the most commonly used metric is air temperature or dry-bulb temperature (T_db_). A standardized method for measuring outdoor T_db_ was developed by Thomas Stevenson in 1864, based on the use of an enclosure to protect meteorological instruments against precipitation and direct heat radiation, while still allowing air to circulate freely around them. These so-called ‘Stevenson Screens’ were modified to their present configuration in 1884 and have been used world-wide since that time to collect temperature and other meteorological data under a standardized set of procedures [11, 12]. For the most part, the use of T_db_ data to evaluate human heat limits has relied on the relationship between air temperatures and heat-related morbidity and/or mortality (e.g. see [13]). Except for the fact that hotter temperatures have been associated with increases in heat-related illnesses and deaths [14, 15], identifying a specific T_db_ heat tolerance limit is not practicable. This is because a variety of other meteorological conditions, most notably humidity and direct solar radiation, affect the way heat is experienced physiologically [16].

Over 100 climate indices have been created to counteract this limitation and to produce measures of heat stress that more accurately reflect the physiological strain endured by people exposed to hot climates [17, 18]. Of these, three appear to be commonly used: wet-bub temperature (Tw), wet-bulb globe temperature (WBGT) and the Heat Index (HI). Each can be used to estimate dangerous levels of heat exposure, as well as the upper limits of heat tolerance—although they are based on somewhat different mixes of climatic measures and/or physiological considerations (See Explanatory Box 1).

**EXPLANATORY BOX 1**

**Computation of Heat Indexes**

USNWS Heat Index

$$\begin{array}{l} \begin{array}{lllllllll} HI=\; & -\;42.379+2.04901523(T)\; & +\;10.14333127(RH)\; & -\;.22475541(T)(RH)\; & -\;.00683783(T^{2})\; & -\;.05481717(RH^{2})\; & +\;.00122874(T^{2})(RH)\; & +\;.00085282(T)(RH^{2})\; & -\;.00000199(T^{2})(RH^{2}) \end{array} \end{array}$$

where T = ambient dry-bulb temperature (°F); RH = relative humidity (integer percentage).

([22])

WBGT

Outdoors: $WBGT=0.7\left(T_{NWB}\right)+0.2\left(T_{G}\right)+0.1\left(T_{DB}\right)$

Indoors: $WBGT=0.7\left(T_{NWB}\right)+0.3\left(T_{G}\right)$

Where: T_NWB_ = natural wet-bulb temperature (^o^C); T_G_ = globe temperature (^o^C); T_DB_ = dry-bulb temperature (^o^C).

([30])

Tw is the lowest temperature to which air can be cooled by the evaporation of water at a constant pressure. It is traditionally measured using a psychrometer, an instrument that consists of two mercury thermometers: a dry thermometer measuring T_db_ and one with a wet wick placed over the thermometer bulb. The temperature on the wet-bulb thermometer will drop relative to the dry bulb depending on the degree to which moisture on the wet wick evaporates into the air [19]. At 100% saturation of air, the Tw will equal T_db_ [19]. Tw was first suggested as a way to measure human heat tolerance by J.B.S.Haldane in 1905 [20].

HI is calculated using an algorithm designed to approximate the ‘apparent temperature’ determined by a complex physiological model involving a large number of human thermal variables, including core temperature, heat production and heat transfer under different combinations of high temperature and humidity [21]. The ‘apparent temperature’ generated by this model is equivalent to the air temperature perceived by an average person who is exposed to a particular combination of heat and humidity. Thus, it translates various air temperatures and air moisture levels into a single scale, measured in the same units as air temperature. The US National Weather Service (USNWS) uses an algorithm [22] that most closely approximates the ‘apparent temperatures’ in Steadman’s original tables [23] to classify heat stress into a series of categories that involve progressively greater risk of heat-related illnesses. Since HI is a perceptual index that involves assumptions about body size and ambient conditions, there often exist problems in its application [24]. However, as a general proxy for heat stress, it has proved sufficiently robust to be a significant predictor of heat-related mortality [25–27] and emergency room visits after a heat wave [28]. It is in this perspective that it seems to be used in research (e.g. [29]).

WBGT is designed to incorporate solar radiation and wind, as well as temperature and humidity, into the quantification of heat stress. Its calculation is based on the natural wet-bulb temperature (T_nwb_), the temperature inside a black globe (T_g_), and T_db_. T_nwb_ is measured by a wet-bulb thermometer that is naturally ventilated and exposed to ambient thermal radiation, thereby quantifying evaporative cooling relative to radiant heat and air currents [24]. T_g_ is measured by a thermometer inside a sealed, matte-black, thin-walled globe 15 cm in diameter, thereby quantifying radiant heat load in direct sunlight [30]. The first use of WBGT to monitor heat stress was based on a series of observations that identified the weather conditions at which disabling heat symptoms began to appear in US Marine Corps recruits training in the hot humid conditions of a South Carolina summer [31].

Table 1 shows that the thresholds (and threshold names) associated with progressively elevated risks of heat-related disorders differ among the three indexes. Because the meteorological variables used in the calculations of these indices also differ, the temperatures (noted in °C) associated with increasing levels of severity are not the same. While HI values themselves might be used as proxies for relative heat stress, the HI ranges associated with the risk categories noted in Table 1 (from the USNWS HI chart, see [32]) are questionable. They appear to be derived from a 1981 review of different heat stress indexes [35] which included a figure that presented, without any explanation for its derivation, a chart nearly identical to the one that is currently used by the USNWS. It identified a HI threshold of 130°F (54°C) as the point at which heatstroke becomes ‘inevitable’. Despite attempts to reevaluate and redefine the HI values associated with risk categories (e.g. 24), the lack of sound scientific evidence for these categories [36] makes accepting the 54°C threshold value seem problematic.

**Table 1.** Thresholds associated with progressively more severe heat stress identified by the HI, WBGT and T_WB_

	HI	WBGT	T_W_
Threshold levels for increasing heat stress	Advice on heat exposure related to likelihood of heat-related disorders (measured in the shade)	Heat safety thresholds related to stress of working or exercising in direct sunlight	Point at which the ability to compensate heat production by heat loss is exceeded, leading to unsustainably high core temperature
	27°C Caution	26°C Moderate stress	
	32°C Extreme caution	30°C High stress	
	41°C Danger	34°C Extreme stress (workability limit)	
	54°C Extreme danger	40°C (survivability limit)	30°C–31°C (compensability limit in hot- humid environments) 25°C–28°C (Compensability limit in hot-dry environments)
	[32]	[33]	[34]

Although each US organization that uses WBGT to establish the advisability of exercise in the heat has a somewhat different set of temperature categories, all seem to set the upper limit for ‘workability’ (i.e. the ability to work or exercise in the heat) at WBGT = 34^o^C (see [37]). This can be distinguished from a ‘survivability’ limit of WBGT ≥ 40°C—the point at which heat exposure causes core body temperature to increase to potentially fatal levels during low-intensity physical activity [33]. One widely used set of WBGT guidelines, so-called threshold value limits (TVLs) [38], indicates that the upper threshold of tolerable heat exposure is not a single value, but diminishes from a WBGT of about 34°C at low workloads to a WBGT of about 26°C at high workloads [39]. A series of laboratory studies designed to monitor heart rate, skin temperature and core temperature during a series of progressively greater levels of heat stress [39, 40] indicate that TVLs generally correspond to the maintenance of a sustainable core body temperature no greater than 38°C [40]. These heat thresholds also have been validated as significant predictors of both heat-related deaths and heat-related illnesses among outdoor workers (e.g. [41],—although both can occur over a wide range of temperatures and relative humidities [30]).

Tw has generated a great deal of interest because it seems to clearly identify a point beyond which human life cannot be sustained. This point (Tw = 35°C) was determined by reference to a physiological model of the maximum temperature of the skin at which heat loss to the environment can occur and a normal core temperature of 37°C can be maintained [42]. When environmental heat and humidity cause this limit to be reached or exceeded, core temperatures increase to deadly levels. Thus, under conditions when the environmental T_db_ > 35°C and Tw ≥ 35°C for more than a few hours, this model predicts that the evaporation of sweat would be unable to cool the surface of the skin, thereby making thermoregulation in the heat impossible [42, 43]. Recently, laboratory studies of young, healthy adults that monitored physiological responses during rest and light exercise under various T_db_ and Tw conditions [34, 44–46] indicated that critical core temperatures (i.e. above 40.2°C) are reached considerably below Tw = 35°C. During rest, thermoregulatory limits in hot dry environments are reached when Tw = 25°C to 28°C, and thermoregulatory limits in hot humid environments are reached when Tw = 30°C to 31°C (average = 30.62°C) [34, 45].

Predictions of future climate conditions indicate that at some point by the end of this century, the heat in many locations around the world will approach or exceed the extreme thresholds of all the indexes indicated in Table 1 [7, 33, 44, 45, 47–50]. Understanding how humans will cope with increasing temperatures as they approach these thresholds can be gauged by studying how humans experience and adjust to high temperatures today. An important first step in this endeavor is to distinguish between outdoor temperatures—those most commonly used to measure heat stress—and temperatures experienced by individuals as they go about their daily lives.

Small, wearable telemetric monitoring devices make it possible to continuously measure the heat and humidity experienced by individuals both during the day, when they are active and at night when they are mostly sedentary and indoors [51–54]. As a result, it is possible to understand how individual variability in heat exposure is affected by behaviors that reflect attitudes, lifestyles, activity schedules, household income, education, residence, and occupation [51, 52, 55, 56]. Studies using continuous monitoring indicate that experienced temperatures and humidity often differ significantly from outdoor measurements made at fixed-site weather stations [52, 53, 57]. Among studies conducted in temperate environments, some indicate that individually experienced temperatures (IET) are lower than outdoor ambient conditions [52, 58], some indicate that IETs are higher than outdoor conditions [54, 59], while others report variable responses, depending on outdoor conditions, and on time spent outdoors versus indoors (where air conditioning generally produced cooler conditions) [51, 56]. On the other hand, studies conducted in hot, humid tropical environments—those that are likely to experience extreme heat events due to global warming [60] —have indicated that indoor heat conditions in low-income housing commonly exceed those that exist outdoors [61–63]. This being the case, it is important to establish the level of severity experienced by the urban poor who currently live in densely packed housing without air conditioning—including perhaps one billion people world-wide who live in informal settlements [64].

## INDOOR CONDITIONS: A CASE STUDY

A study that Dr. Barun Mukhopadhyay (of the Indian Statistical Institute) and I conducted among residents of slum dwellings of Kolkata during a hot humid summer [61, 65, 66] shows just how closely indoor heat conditions approach the upper thresholds indicated in Table 1. T_db_, HI, WBGT and Tw were monitored during 24-hour periods in 123 dwellings between May and August in 2019 (see [61] for details). Most of the dwellings (74%) consisted of a single room with cement or brick walls and roofs of corrugated iron (27%), cement (23%) or terracotta tiles (50%). Construction materials contributed to the absorption and retention of heat via solar radiation, which was exacerbated by indoor cooking (on kerosine or coal cookstoves), appliances (refrigerators, televisions and radios), and body heat generated by as many as 7 residents (average = 3.3). None of the dwellings had air conditioning. Windows were small, sometimes absent (6%) and along with doors, were invariably closed at night. Since the room size was small (most were less than 200 ft^**2**^, see Explanatory Box 2), heat became intense during the day and did not cool off appreciably at night. While all dwellings had overhead fans, these provide little relief in hot humid conditions [67].

**EXPLANATORY BOX 2**

Example of a dwelling included in the Kolkata heat study.

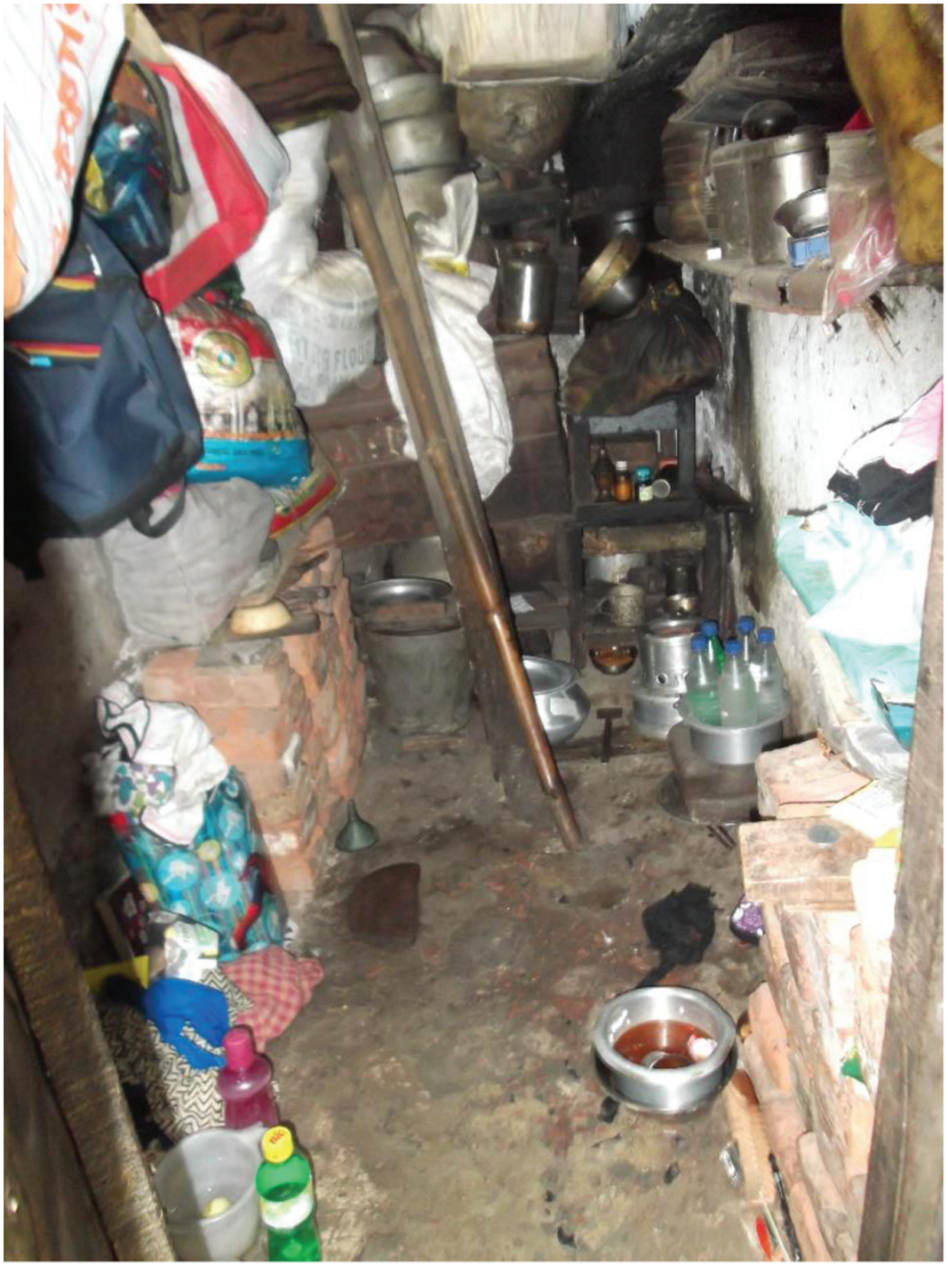

The sleeping area in this small dwelling is off to the left. Cooking area is on the floor to the right. This small unit did not have windows.

**Figure 1.**
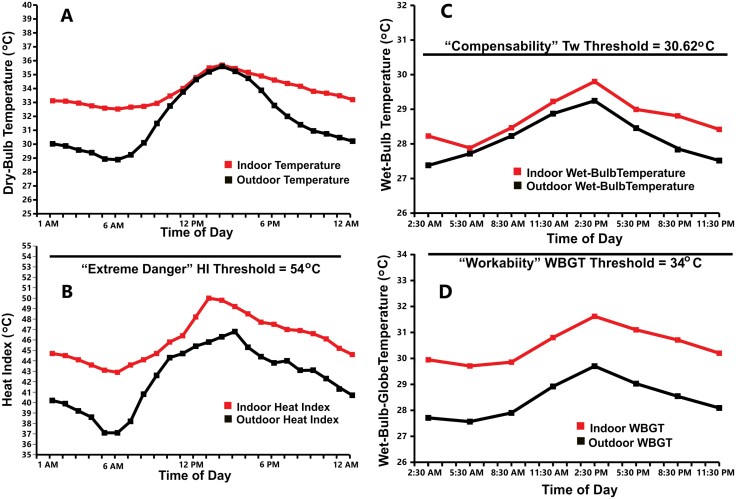
(A—D). Hourly measurements of indoor heat conditions in Kolkata slum dwellings compared to outdoor conditions during May 2019. A. Hourly indoor and outdoor dry-bulb temperature. B. Hourly indoor and outdoor HI measurements relative to ‘extreme danger’ threshold (HI = 54°C, see [32]). C. Indoor and outdoor wet-bulb temperature measurements (at 4-hour intervals) relative to ‘compensability’ threshold in not humid environments (Tw = 30.62°C, see [34]). D. Indoor and outdoor wet-bulb globe temperature measurements (at 4-hour intervals) relative to ‘workability’ threshold (WBGT = 34°C, see [33]). In the absence of solar radiation, indoor measurements corresponded to ‘simplified’ WBGT (= 0.7(TNWB) + 0.3(TG), see Explanatory Box 1 and [30]). Means and standard deviations of all measurements are available in Supplementary Table 1.

Figure 1A–D shows the average values for indoor T_db_, HI, WBGT and Tw recorded throughout the day compared to outdoor values during the hottest month (May). While indoor T_db_ are 3°C to 4°C above outdoor temperatures overnight and equivalent to outdoor temperatures during the hottest part of the day (Fig. 1A), the indoor humidity in Kolkata is reversed (lower than outdoor humidity overnight, but higher than outdoor humidity during the hottest part of the day). Thus, indoor measurements of HI, WBGT and Tw that include both temperature and humidity in their calculation were considerably above outdoor measurements during most of the daytime—but particularly at night. While the average indoor HI during the hottest time of the day in May (50°C) never reached the putative ‘extreme danger’ threshold of 54°C (Fig. 1B), it *was* above 52°C (130°F) for as many as 6 hours during the day in 3 of the 11 dwellings monitored during that month (27.3%), and in 24 of the 123 dwellings monitored over the summer (19.5%). In addition, the indoor HI exceeded 41°C (a level labeled as being ‘dangerous’. See Table 1) at some point during the day in almost every slum dwelling monitored during the summer (120 out of 123 = 97.6%). Likewise, Fig. 1C shows that average indoor Tw during the hottest time of the day recorded in 11 dwellings in May (29.8°C) approached, but did not exceed, the 30.62°C limit of compensability in hot humid climates [45]. However, during the course of the summer, 19 of the 123 households that were monitored (15.4%), experienced indoor Tw above 30°C for between 2 and 6 hours during the 24-hour study period. Fig. 1D shows that indoor WBGT during the hottest time of the day in May (31.7°C), while above outdoor levels, did not approach the 34°C threshold for ‘workability’ in hot humid environments [39], let alone the ‘survivability’ threshold of 40°C [33]—although it was above 32°C in 3 of the 11 dwellings monitored during May for 2 to 3 hours during the day, and in about 25% of the 123 dwellings monitored over the summer.

**Figure 2.**
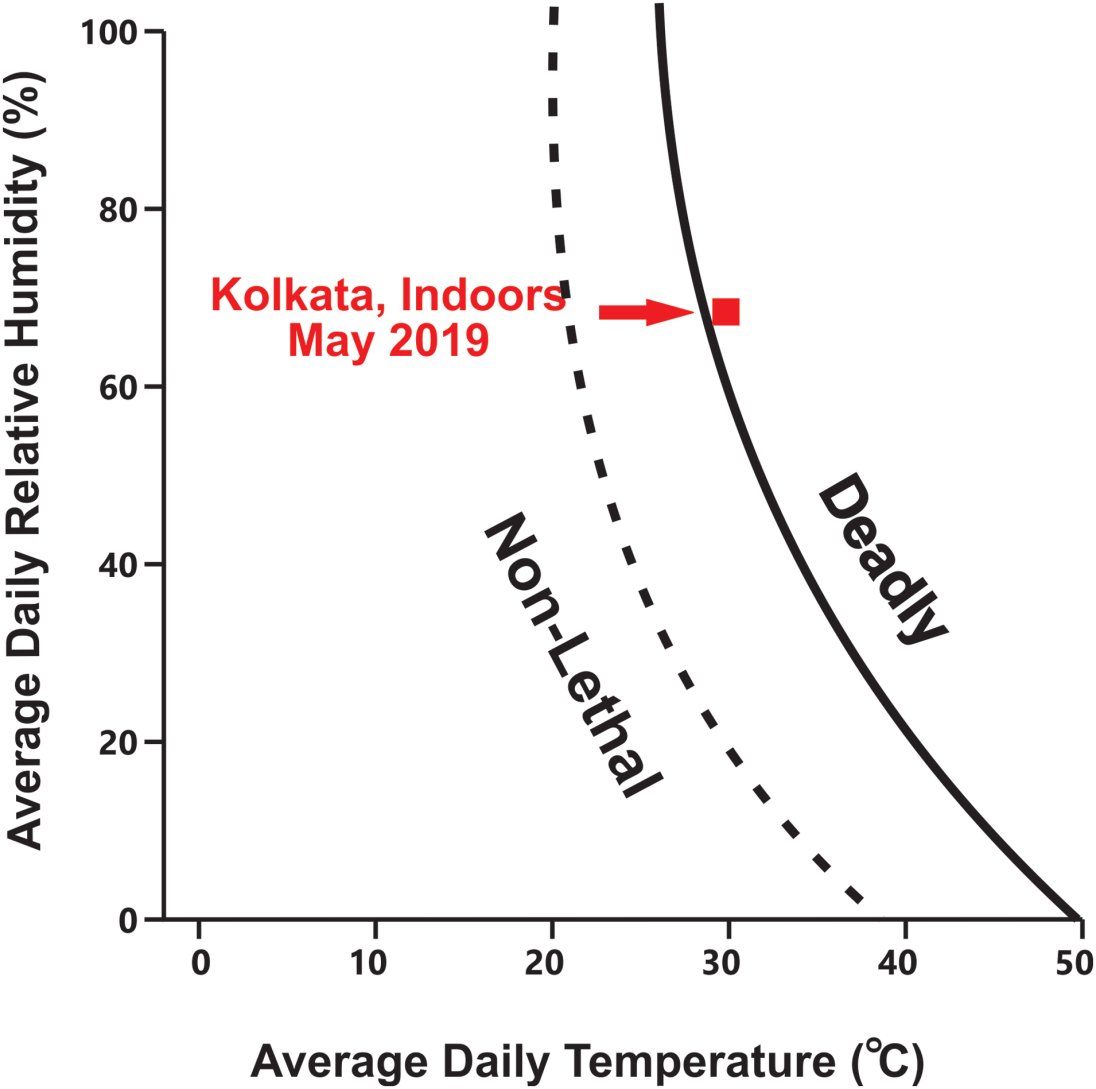
Average indoor temperature and relative humidity in Kolkata slum dwellings during May 2019 relative to threshold between ‘deadly’ and ‘non-deadly’ conditions (redrawn from Mora *et al*. [47], with permission from Springer Nature).

**Figure 3.**
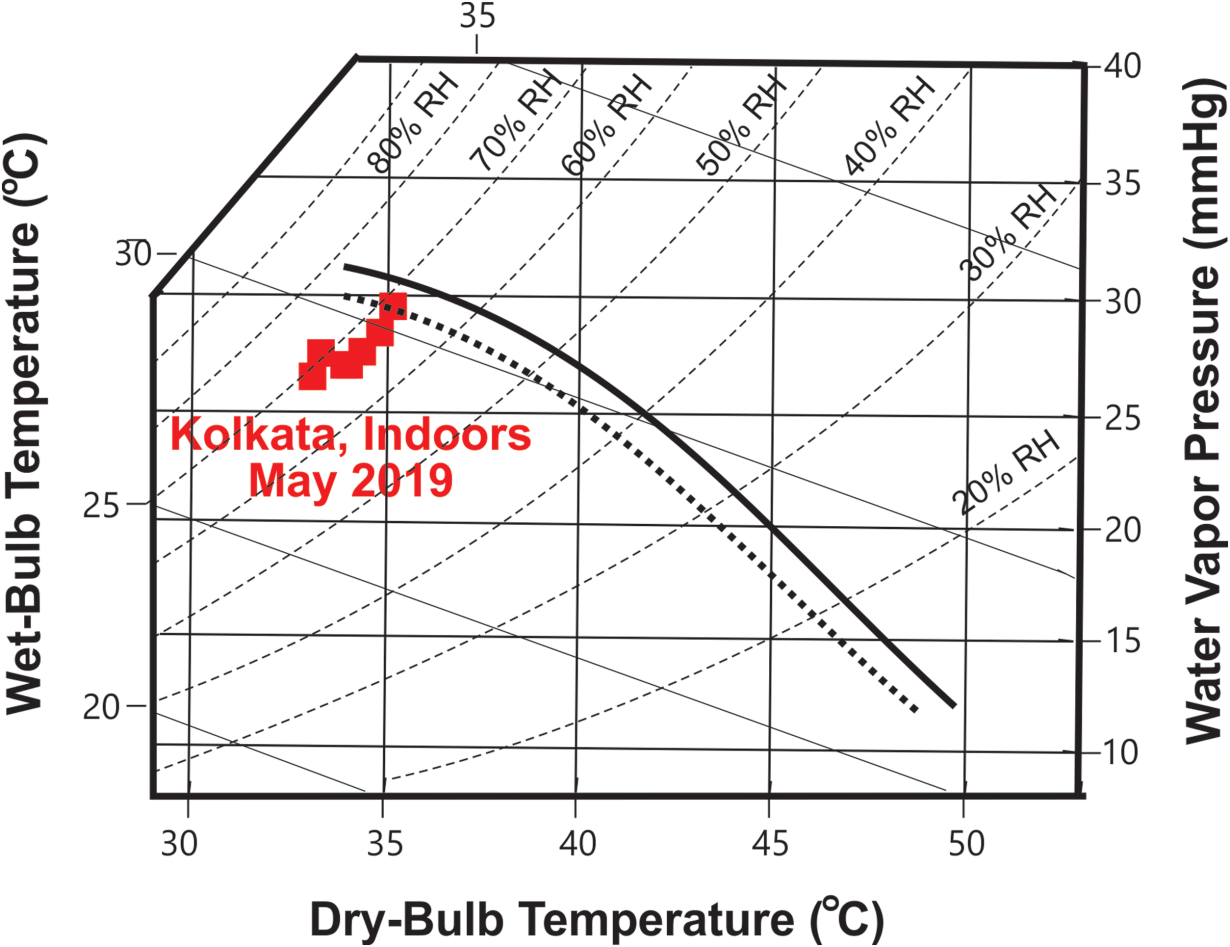
Indoor conditions in Kolkata slum dwellings during May 2019 relative to empirically determined critical environmental limits based on observations of healthy young adults exposed to different levels of heat and humidity during minimal exercise. The dark solid line running from upper left to lower right represents the threshold between compensable and non-compensable conditions. The dark dashed line running below it represents the lower bounds of the 95% confidence interval of the threshold between compensable and non-compensable conditions. (Redrawn from Cottle *et al*. [44], with permission from the American Physiological society). Kolkata data points represent 4-hour averages of Tw, Tdb, RH and water vapor pressure.

Comparisons with other studies provide an assessment of the severity of the indoor heat stress endured by slum inhabitants. Figure 2 shows that the average indoor heat and humidity in Kolkata during May 2019 was slightly above the ‘deadly’ boundary based on heat-related deaths during 783 specific heat events in 164 cities across 36 countries [47]. The ‘deadly’ boundary shown is determined by Support Vector Machines (classification models based on machine learning algorithms) that identified monthly average temperature and humidity as the conditions best able to differentiate between deadly and non-deadly events. Subsequently, this algorithm identified a temperature and humidity ‘boundary’ that maximizes the probability of deadly events falling on one side and non-deadly episodes on the other [47]. Figure 3 shows that indoor conditions during the hottest time of the day in May were near the 95% lower confidence level for the boundary between compensable and uncompensable heat [44]. This ‘boundary’ was determined by exposing heathy young adults to different heat and humidity levels while at rest [44].

In our study, both outdoor and indoor conditions ameliorated somewhat during June, July and August—although conditions like those recorded in May occurred occasionally over the succeeding months. Nevertheless, the duration of exposure to extreme indoor heat never exceeded 6 hours during the 24-hour monitoring period—and thus was not long enough to become life threatening, at least relative to Tw thresholds [44]. On the other hand, each of these dwellings was inhabited by at least one elderly person (age ≥ 65 years)—an age group that is likely to experience severe physiological strain at lower heat levels than those noted by the extreme heat thresholds in each of the indices [68, 69].

## COPING WITH EXTREME HEAT

These data indicate that some people are already experiencing, and surviving in, conditions in the middle to upper range of heat thresholds shown in Table 1—and, in some parts of the world, are even exposed to outdoor conditions that approach the limits of heat tolerance [7]. Thus, it is easy to understand why a large body of ‘heat hazard response’ studies has focused on identifying how human populations cope with and survive in, hot environments (see [70–72] for reviews). For the most part, these studies have focused on behavioral/cultural, infrastructural/technological, institutional (e.g. so-called ‘heat action’ plans), and/or ecosystem-based strategies [67], implemented by governments or individuals [70]. Predictive modeling suggests that these strategies will have a positive effect in the future, although not all models predict the same outcome [73].

In wealthy countries, these heat response strategies have already met with considerable apparent success [74]. For example, mortality reported in successive heat waves in the USA fell considerably between 1974 and 1998 [75], continuing through 2005 [76]—although this trend appears to be diminishing due to variable changes in morality in different age groups [77]. In other parts of the world, the incomplete recording of heat-related mortality (e.g. [78]), prevents a complete understanding of whether, or the extent to which, these heat hazard response initiatives have been effective. In developing countries, institutional approaches are less likely to be implemented, and most people rely on behavioral/cultural responses [72]. Individual behavioral modifications (wearing lighter clothing, drinking liquids, etc) may be relatively easy for most people to at least try; but the extent to which they can provide significant relief—particularly in extremely hot environments—may not be great. Even the use of fans provides only minimal relief, particularly in hot humid environments [67]. Thus, while not to be discounted, the effectiveness of such strategies is limited [66], leaving large populations in poorer countries at much greater risk for adverse heat effects compared to people in wealthy countries [79].

One commonly discussed technological response to increasing global temperatures is air conditioning [80]. This appears to be based on both obvious reductions in heat and humidity produced by air conditioners [81], and on studies conducted in the USA and other wealthy countries that seem to show an advantage to remaining indoors on hot days [51, 54], presumably in an air-conditioned environment [52, 55, 56, 58, 59]. However, the use of air conditioning may not always be associated with lower indoor compared to outdoor temperatures [82]. In the southwest USA, for example, it is estimated that 97% of all occupied housing units have air conditioning [83]. But, even in this location where outdoor temperatures commonly exceed 40^o^C during summer, indoor heat stress is affected by the way people use air conditioning. In low-income households located in the Phoenix area, the use of air conditioning is negatively affected by the cost of electricity and repairing broken units [84]. A 2014 survey of Maricopa County residents found that 16% of the respondents with an air-conditioning unit at home did not use their unit because of cost, and another 4% stated that their air-conditioning unit was broken [85]. Consequently, low-income individuals who lack air conditioning, who use low-efficiency air-conditioning equipment, or who lack funds to run a unit frequently experience high indoor temperatures [86]. This is reflected in a study of heat-related deaths among elderly women in Maricopa County between 2006 and 2016. An air-conditioning unit was present in 73.2% of the dwellings occupied by women aged ≥ 65 years who died indoors, but was not functioning in 46.5%, and not in use in an additional 36.6% [87]. Missing from these associations, unfortunately, is any information on just how hot indoor temperatures became with infrequent or inefficient air conditioner use.

If the use of air conditioning is going to effectively counteract future global temperature increases, it will have to solve several important problems. First, the energy necessary to support extensive air conditioning can easily overload existing electricity capacities and therefore increase the risk of electricity power cuts [88], particularly in poorer countries [89]. Additionally, the heat ejected by air conditioners contributes to the Urban Heat Island effect and directly affects outdoor thermal comfort [90]. Relying on air conditioning to counteract future global heat stress also ignores the expense of purchasing an air conditioning unit, the expense of maintenance and the expense of running it. Thus, in many countries with already warm climates, increases in the use of air conditioning are predicted to reflect income disparities—with wealthier people far more likely to install air conditioning than those who are poorer—creating serious inequities in adoption [91]. Even though several solutions to this problem have been offered, including the development and use of new cooling technologies [92, 93] and climate-sensitive building and urban design [88], it may not be realistic to presume that technology will solve the problem of excessive heat exposure for the billion-plus people who have been estimated to live in informal urban settlements [64], plus those living in ‘formal’ slum settlements, as in Kolkata.

Interestingly, the ‘heat hazard response’ literature generally ignores biological adjustments [70]—except perhaps to worry about whether the extensive use of air conditioning will render people unable to adjust to outside environments [90]. This is surprising since the physiological changes that occur during exposure to the heat make it possible to increase heat loss by increasing blood flow to the periphery and by increasing sweat rate, thereby lowering core temperature, reducing cardiovascular strain, and permitting physical work capacity to be maintained or even increased [94, 95]. By enhancing heat tolerance, physiological acclimatization to the heat also alters the subjective assessment of thermal comfort and makes people more accustomed to, and more tolerant of, warmer environments [95, 96]. Consequently, people living in areas with generally higher day-to-day ambient temperatures or who are more frequently exposed to temperature extremes have a reduced mortality rate when exposed to heat waves compared to people living in more temperate climates [97–99]. Likewise, studies of heat-related illnesses frequently indicate that hospitalizations are associated with a *lack* of acclimatization [100–102]. Thus, heat loads that might be uncompensable (i.e. heat production exceeding heat loss potential) before acclimatization become compensable (heat production matched by heat loss) afterwards [103].

The degree to which individuals can adjust to high environmental temperatures is related to the intensity, frequency and number of heat exposures, in addition to their duration [94, 104]. Furthermore, the level of acclimatization that can be achieved is known to vary, depending on age [105], gender [106] and co-morbidities [16]. Thus, physiological acclimatization to the heat is not an ‘open-ended’ process, and heat tolerance can be breeched even among those who are routinely exposed to hot conditions and who therefore are more likely to achieve higher levels of acclimatization [16].

An important evolutionary question is whether there is a genetic component associated with *individual* variability (within age, gender and health categories) in the capacity to adjust to the heat and whether in the context of increasing global heat, such variability might be the object of natural selection. Many attempts to address this question—based on the hypothesis that there might be genetic differences in heat tolerance between populations—were conducted in the middle decades of the 20th century. These studies focused on differences between ethnic groups that were indigenous to hot environments and control groups from temperate climates [107, 108]. Considerable interest was generated by the possibility that body form might be beneficial for heat exchange in hot environments. This was particularly true after an early study indicated that groups indigenous to hot environments had higher surface-area-to-mass ratios, thereby permitting greater heat loss, compared with those living in colder environments [109]. However, research conducted some 40 years later indicated that the surface-area-to-mass ratios of tropical populations had declined, likely due to improvements in nutrition [110]—a change that draws into question whether population variability in body form indicates anything about population genetic differences in climate adaptation [111, 112].

This is not to say that variations in body form, whether from genetic or environmental causes, are unrelated to physiological responses in the heat. During exercise in the heat, several studies indicated that smaller individuals (i.e. those with higher surface-area-mass-ratios) performed better than larger individuals—particularly in hot humid conditions when heat dissipation mechanisms were at their limit [113–116]. However, some of this apparent advantage appears related to lower levels of metabolic heat production among smaller compared to larger individuals who were exercising at the same rate [117]. At equivalent metabolic heat loads, body mass is negatively correlated with changes in rectal temperature [118–120], leading to the conclusion that larger people (i.e. those with lower surface-area-to-mass ratios) experience lower increases in core temperature during exercise in hot conditions, presumably because a larger body mass allows for a greater distribution of internal heat [121]. One conclusion that has been drawn from these studies is that the influence that surface-area-to-mass ratios may have on exercise in hot conditions is variable, depending on the type of exercise, the exercise intensity and the environmental conditions under which exercise occurs [122].

Body form also may influence the types of thermoregulatory responses that people make during the acclimatization process. High whole-body sweat rate responders tend to be larger individuals with low surface-area-body-mass ratios, high heart rate responders tend to be smaller individuals with high surface-area-body-ratios, and high rectal temperature responders tend to be fitter with lower percentages of body fat [122, 123]. This has led to the suggestion that individuals will acclimatize according to the pathway that best suits their body type [124].

It appears that some, so-called ‘heat tolerant’, individuals are able to adjust to heat without serious side effects, while other, so-called ‘heat intolerant’, individuals are more likely to suffer from heat exhaustion, heat stroke or other serious heat-related conditions [125]. Each type can be distinguished using controlled laboratory heat stress tests [126]. Physiologically, the characteristics of heat-tolerant individuals will vary, suggesting that there is no single thermoregulatory response pattern that characterizes all such individuals [127]. However, it has been suggested that the distinction between ‘heat tolerant’ and ‘heat intolerant’ individuals may also involve a genetic component, associated with the differential transcription of heat shock proteins [128–131]. Heat shock proteins are activated upon exposure to increases in heat at the cellular level to prevent cell damage and death [132] and reflect a change in gene expression [133]. Thus, it is possible that epigenetic mechanisms may underlie both potentially advantageous and potentially deleterious transcriptional responses during acclimatization, producing long-lasting changes in DNA methylation near the promotor regions of genes involved in basic cell function [131, 134]. If both advantageous and deleterious epigenetic changes can be inherited [131], this could produce an important foundation for the action of natural selection in hot environments. However, most evidence for the inheritance of epigenetic changes occurs in non-human animals and is presently a highly controversial topic with reference to humans [135]. Epigenetic changes notwithstanding, further research will be required to determine whether there exist inherited differences in the transcription of heat shock proteins that affect overall adaptation to the heat, or whether advantageous and deleterious responses are merely transitory states [136].

## Conclusions

Predicting whether people will—or will not—be able to cope with warmer climates in areas of the world already experiencing hot summers requires a more complete understanding of how people experience heat today. Thus far, only a small number of studies recording IETs have been conducted in the general population, and most have occurred in high-income countries (e.g. [51, 52, 56, 58, 137]). Further global studies designed to continuously monitor the heat conditions—particularly among vulnerable subpopulations, such as the elderly—living under extreme conditions (similar to the study described in [65]) will be of considerable importance.

Research also is needed to record the level of indoor heat experienced by individuals with and without air conditioning. A major conclusion from this short review is that indoor heat in low-income, densely packed urban settlements—particularly in the hot-humid tropics—may already be at or near putative uncompensable levels, producing a significant burden on people living under these conditions. However, these problems are not confined to populations living in the hot tropics. Even in the USA, indoor heat in poor households in the Southwest, as well as in low to middle-income housing in urban areas, may regularly exceed levels that are potentially harmful to health [82].

Evaluating the role of biological adjustments to the heat is also incomplete. It will be important to understand the potential genetic component related to heat shock protein activation. However, a more complete picture of human acclimatization potential could be achieved if additional information were available on the physiological responses of populations indigenous to hot climates—particularly hot, humid climates—as they go about their daily lives. In this regard, further studies will be necessary to determine if the long-term acclimatization that occurs among indigenous groups exposed to hot conditions since birth is similar to, or even exceeds, the heat adjustments that can be produced during controlled laboratory acclimation studies conducted on people living in temperate environments. For example, elderly Kolkata slum residents who live their entire lives in a hot humid tropical environment endure indoor heat conditions that seem to be beyond levels that can be tolerated by apparently unacclimatized elderly in controlled studies [138, 139]. Thus, it seems reasonable to investigate the physiological responses of elderly (and others) exposed to long-term heat to determine whether there are potentials that remain to be quantified.

## Supplementary Material

eoae015_suppl_Supplementary_Materials
