# Supplementary material for: Coping with extreme heat: current exposure and implications for the future
Source: Evol Med Public Health. 2024 Aug 22;12(1):156–68. doi: 10.1093/emph/eoae015 (PMC11445678; doi:10.1093/emph/eoae015)
Supplement: eoae015_suppl_Supplementary_Materials [file eoae015_suppl_supplementary_materials.docx]

Supplemental Table 1. Means and Standard Deviations of Temperature Measurements. All data are presented in ^o^C

| **Time** | **Outside Tdb** | **Standard Deviation** |  | **Inside Tdb** | **Standard Deviation** |
| --- | --- | --- | --- | --- | --- |
| **1:00 AM** | **30.03** | **0.62** |  | **33.12** | **0.52** |
| **2:00 AM** | **29.87** | **0.66** |  | **33.09** | **0.58** |
| **3:00 AM** | **29.58** | **0.63** |  | **32.95** | **0.58** |
| **4:00 AM** | **29.39** | **0.68** |  | **32.75** | **0.67** |
| **5:00 AM** | **28.94** | **0.55** |  | **32.59** | **0.69** |
| **6:00 AM** | **28.89** | **0.43** |  | **32.53** | **0.52** |
| **7:00 AM** | **29.24** | **0.89** |  | **32.66** | **0.60** |
| **8:00 AM** | **30.10** | **0.99** |  | **32.72** | **0.80** |
| **9:00 AM** | **31.49** | **1.07** |  | **32.94** | **0.90** |
| **10:00 AM** | **32.75** | **1.01** |  | **33.46** | **1.14** |
| **11:00 AM** | **33.87** | **0.89** |  | **34.00** | **0.96** |
| **12:00 PM** | **34.76** | **0.78** |  | **34.79** | **1.17** |
| **1:00 PM** | **35.08** | **1.59** |  | **35.48** | **1.33** |
| **2:00 PM** | **35.59** | **1.03** |  | **35.68** | **1.43** |
| **3:00 PM** | **35.24** | **0.94** |  | **35.44** | **1.31** |
| **4:00 PM** | **34.74** | **0.98** |  | **35.16** | **1.10** |
| **5:00 PM** | **33.86** | **1.00** |  | **34.90** | **0.77** |
| **6:00 PM** | **32.78** | **0.81** |  | **34.61** | **0.60** |
| **7:00 PM** | **32.00** | **0.55** |  | **34.36** | **0.49** |
| **8:00 PM** | **31.40** | **0.41** |  | **34.16** | **0.72** |
| **9:00 PM** | **30.95** | **0.38** |  | **33.80** | **0.43** |
| **10:00 PM** | **30.75** | **0.41** |  | **33.66** | **0.49** |
| **11:00 PM** | **30.47** | **0.44** |  | **33.39** | **0.47** |
| **12: 00 AM** | **30.22** | **0.49** |  | **33.20** | **0.46** |
|  |  |  |  |  |  |
| **Time** | **Outside HI** | **Standard Deviation** |  | **Inside HI** | **Standard Deviation** |
| **1:00 AM** | **40.17** | **3.02** |  | **44.66** | **2.14** |
| **2:00 AM** | **39.93** | **3.07** |  | **44.52** | **2.23** |
| **3:00 AM** | **39.16** | **2.69** |  | **44.15** | **2.24** |
| **4:00 AM** | **38.63** | **2.69** |  | **43.63** | **2.58** |
| **5:00 AM** | **37.06** | **2.08** |  | **43.14** | **2.37** |
| **6:00 AM** | **37.07** | **1.57** |  | **42.85** | **1.64** |
| **7:00 AM** | **38.22** | **3.02** |  | **43.62** | **2.05** |
| **8:00 AM** | **40.85** | **3.32** |  | **44.06** | **2.73** |
| **9:00 AM** | **44.30** | **0.85** |  | **44.68** | **2.30** |
| **10:00 AM** | **44.73** | **1.24** |  | **45.83** | **2.31** |
| **11:00 AM** | **45.35** | **1.63** |  | **46.44** | **1.56** |
| **12:00 PM** | **45.79** | **2.06** |  | **48.21** | **2.64** |
| **1:00 PM** | **46.30** | **2.10** |  | **49.96** | **2.68** |
| **2:00 PM** | **46.80** | **2.33** |  | **49.75** | **3.01** |
| **3:00 PM** | **45.31** | **1.81** |  | **49.21** | **2.42** |
| **4:00 PM** | **44.38** | **2.35** |  | **48.49** | **1.83** |
| **5:00 PM** | **42.62** | **2.19** |  | **47.68** | **1.08** |
| **6:00 PM** | **43.83** | **1.78** |  | **47.49** | **1.37** |
| **7:00 PM** | **43.98** | **1.23** |  | **46.98** | **1.06** |
| **8:00 PM** | **43.14** | **1.09** |  | **46.92** | **1.41** |
| **9:00 PM** | **43.09** | **0.90** |  | **46.59** | **0.85** |
| **10:00 PM** | **42.28** | **1.85** |  | **46.10** | **1.61** |
| **11:00 PM** | **41.33** | **2.29** |  | **45.23** | **2.00** |
| **12: 00 AM** | **40.70** | **2.88** |  | **44.58** | **2.01** |
|  |  |  |  |  |  |
| **Time** | **Outside WBGT** | **Standard Deviation** |  | **Inside WBGT** | **Standard Deviation** |
| **2:30 AM** | **27.71** | **0.64** |  | **29.95** | **0.72** |
| **5:30 AM** | **27.56** | **0.56** |  | **29.63** | **0.61** |
| **8:30 AM** | **27.90** | **0.66** |  | **29.85** | **0.63** |
| **11:30 AM** | **28.92** | **0.34** |  | **30.80** | **0.55** |
| **2:30 PM** | **29.70** | **0.62** |  | **31.62** | **0.89** |
| **5:30 PM** | **29.03** | **0.29** |  | **31.10** | **0.39** |
| **8:30 PM** | **28.55** | **0.24** |  | **30.71** | **0.34** |
| **11:30 PM** | **28.09** | **0.48** |  | **30.20** | **0.61** |
|  |  |  |  |  |  |
|  |  |  |  |  |  |
| **Time** | **Outside Tw** | **Standard Deviation** |  | **Inside Tw** | **Standard Deviation** |
| **2:30 AM** | **27.38** | **0.92** |  | **28.23** | **0.93** |
| **5:30 AM** | **27.72** | **0.84** |  | **27.88** | **0.75** |
| **8:30 AM** | **28.23** | **0.92** |  | **28.49** | **0.66** |
| **11:30 AM** | **28.87** | **0.56** |  | **29.23** | **0.46** |
| **2:30 PM** | **29.25** | **0.55** |  | **29.81** | **0.58** |
| **5:30 PM** | **28.45** | **0.56** |  | **28.95** | **0.34** |
| **8:30 PM** | **27.84** | **0.61** |  | **28.81** | **0.16** |
| **11:30 PM** | **27.52** | **0.78** |  | **28.42** | **0.79** |
